# Supplementary material for: Heme Oxygenase-1 Exerts Antiviral Activity against Hepatitis A Virus In Vitro
Source: Pharmaceutics. 2021 Aug 9;13(8):1229. doi: 10.3390/pharmaceutics13081229 (PMC8401830; doi:10.3390/pharmaceutics13081229)
Supplement: Supplementary file 1 [file pharmaceutics-13-01229-s001.zip › pharmaceutics-1328541-supplementary.pdf]

Supplementary Material

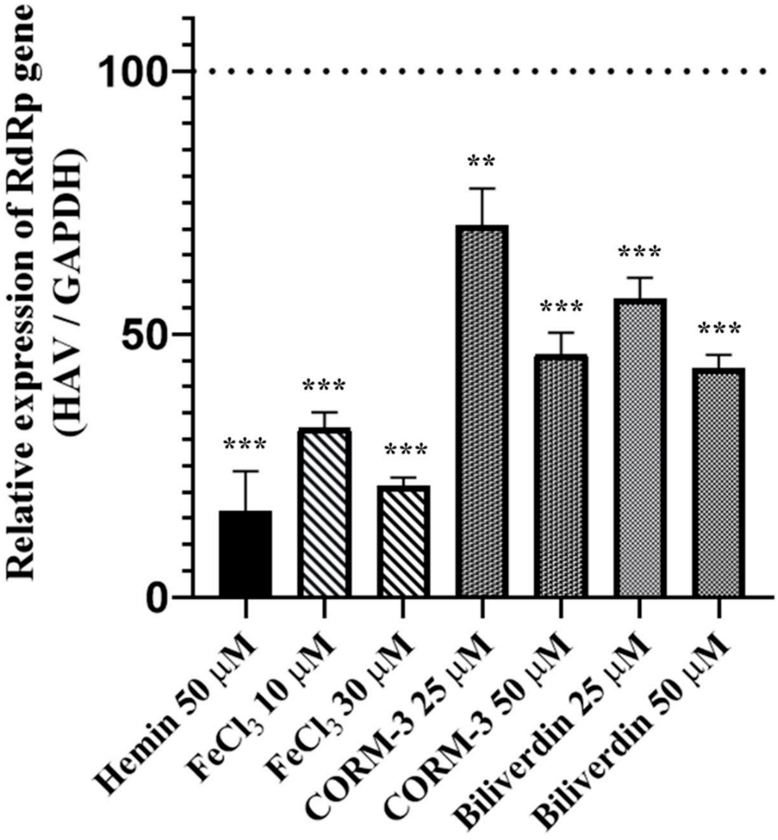

Figure S1. Changes in the amount of HAV RNA-dependent RNA polymerase gene. FRhK-4 cells infected with HAV were treated for hemin, $FeCl_3$, CORM-3, and biliverdin. HAV RdRp genes which play a key role in the replication of virus were reduced by HO-1 induction and three representative metabolites of heme that are produced through the enzymatic activity of HO-1. Data are presented as the mean ± standard deviations (SD) of three independent experiments. Data are presented as the mean ± standard deviations (SD) of at least 3 independent experiments. ***P*<0.01; ****P*<0.001.

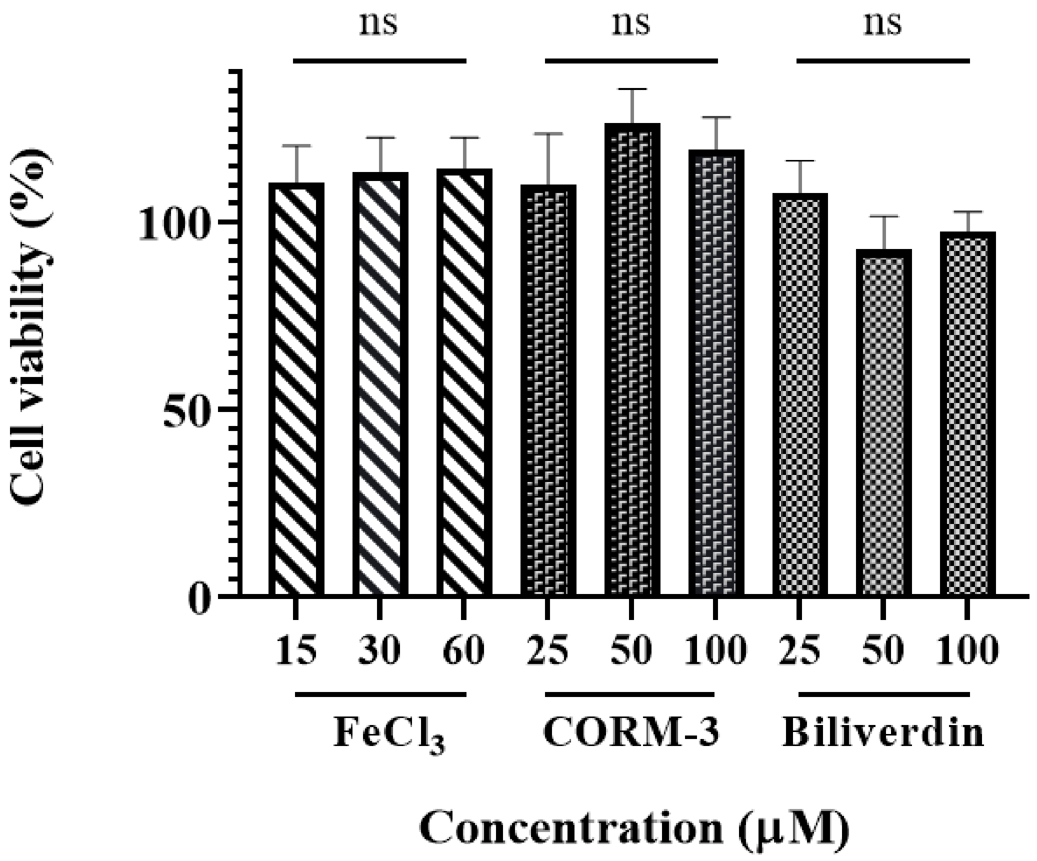

Figure S2. Cytotoxicity assay for $FeCl_3$, CORM-3, and biliverdin. Cell viability percentages (compared to MOCK-treated cells) of FRhK-4 cells on 72 h post-treatment with the indicated concentrations of chemicals. Data are presented as the mean ± standard deviations (SD) of at least three independent experiments. ns, non-significant.
